# Transcriptional analysis of defense mechanisms in upland tetraploid switchgrass to greenbugs

**DOI:** 10.1186/s12870-017-0998-2

**Published:** 2017-02-16

**Authors:** Teresa Donze-Reiner, Nathan A. Palmer, Erin D. Scully, Travis J. Prochaska, Kyle G. Koch, Tiffany Heng-Moss, Jeffrey D. Bradshaw, Paul Twigg, Keenan Amundsen, Scott E. Sattler, Gautam Sarath

**Affiliations:** 1 Department of Biology, West Chester University of Pennsylvania, West Chester, PA 19383 USA; 2 Wheat, Sorghum, and Forage Research Unit, USDA-ARS, 251 Filley Hall, East Campus, UNL, Lincoln, NE 68583-0937 USA; 3 Stored Product Insect and Engineering Research Unit, USDA-ARS, Manhattan, KS 66502 USA; 4 Department of Entomology, University of Nebraska-Lincoln, Lincoln, NE 68583-0816 USA; 5 Biology Department, University of Nebraska-Kearney, Kearney, NE 68849 USA; 6 Department of Agronomy and Horticulture, University of Nebraska-Lincoln, Lincoln, NE 68583-0915 USA; 7 Present address: North Central Research Extension Center, North Dakota State University, South Minot, ND 58701 USA

**Keywords:** Switchgrass, Aphids, GB, ROS, RNA-Seq, Metabolites, Network, Plant defense, Pipecolic acid, Chlorogenic acid

## Abstract

**Background:**

Aphid infestation of switchgrass (*Panicum virgatum*) has the potential to reduce yields and biomass quality. Although switchgrass-greenbug (*Schizaphis graminum;* GB) interactions have been studied at the whole plant level, little information is available on plant defense responses at the molecular level.

**Results:**

The global transcriptomic response of switchgrass cv Summer to GB was monitored by RNA-Seq in infested and control (uninfested) plants harvested at 5, 10, and 15 days after infestation (DAI). Differentially expressed genes (DEGs) in infested plants were analyzed relative to control uninfested plants at each time point. DEGs in GB-infested plants induced by 5-DAI included an upregulation of reactive burst oxidases and several cell wall receptors. Expression changes in genes linked to redox metabolism, cell wall structure, and hormone biosynthesis were also observed by 5-DAI. At 10-DAI, network analysis indicated a massive upregulation of defense-associated genes, including NAC, WRKY, and MYB classes of transcription factors and potential ancillary signaling molecules such as leucine aminopeptidases. Molecular evidence for loss of chloroplastic functions was also detected at this time point. Supporting these molecular changes, chlorophyll content was significantly decreased, and ROS levels were elevated in infested plants 10-DAI. Total peroxidase and laccase activities were elevated in infested plants at 10-DAI relative to control uninfested plants. The net result appeared to be a broad scale defensive response that led to an apparent reduction in C and N assimilation and a potential redirection of nutrients away from GB and towards the production of defensive compounds, such as pipecolic acid, chlorogenic acid, and trehalose by 10-DAI. By 15-DAI, evidence of recovery in primary metabolism was noted based on transcript abundances for genes associated with carbon, nitrogen, and nutrient assimilation.

**Conclusions:**

Extensive remodeling of the plant transcriptome and the production of ROS and several defensive metabolites in an upland switchgrass cultivar were observed in response to GB feeding. The early loss and apparent recovery in primary metabolism by 15-DAI would suggest that these transcriptional changes in later stages of GB infestation could underlie the recovery response categorized for this switchgrass cultivar. These results can be exploited to develop switchgrass lines with more durable resistance to GB and potentially other aphids.

**Electronic supplementary material:**

The online version of this article (doi:10.1186/s12870-017-0998-2) contains supplementary material, which is available to authorized users.

## Background

Plants respond to insect herbivory with refined and vigorous innate immune responses that trigger a plethora of inducible defenses, which can include localized cell death, structural fortifications such as cell-wall strengthening (via callose, lignin, and cellulose deposition), biochemical and molecular associated defenses [1, 2], and the reallocation of plant nutrients away from the feeding site, which negatively impacts plant nutritional quality [3, 4]. These innate immune responses to herbivory are stimulated by tissue damage and by removal of nutrients, the degree of which is significantly affected by plant genotype. These resultant responses have been categorized as resistant (negative effects on pest biology and/or behavior), susceptible, and tolerant (an ability to overcome damage caused by pests). The degree to which plants can overcome biotic stresses, including insect feeding, has been linked to plant resistance genes (*R*-genes) [5–7]. For example, 33 NBS-LRR *R* genes were induced in wheat in response to gall midge infestation and were linked to the elicitation of defense responses [8, 9]. In addition to possible interactions with plant *R*-genes, aphid herbivory can elicit other short and long term changes to plant physiology that shape the fitness of the host and can contribute to its ability to overcome herbivory.

Aphids, such as greenbugs (*Schizaphis graminum*; GB), are predominately phloem-feeders, that negatively affect plant fitness and health by removing nutrients and secreting toxic salivary compounds into phloem during feeding. Aphid herbivory can therefore lead to extensive plant damage, such as yellowing and/or death of leaves, reductions in plant vigor, and ultimately reduction in yields [10, 11]. Aphids also excrete considerable amounts of sugars through their honeydew, providing a substratum for fungal and bacterial colonization of leaf surfaces which could result in further direct or indirect injury to the plant [12–14].

Initial responses to aphid herbivory appear to include calcium, cell wall kinases, and reactive oxygen species (ROS) responsive signaling networks [2, 7, 15, 16], with later responses affecting photosynthesis and growth. Resistant plants appear to activate some defensive responses, but these are generally not sustained, since the pest does not feed or reproduce on such plants [17, 18]. In susceptible plants, a vigorous defensive response is generally initiated, but the plant is unable to sustain this response and ultimately dies [18, 19]. Tolerant plants, in contrast, appear to either have a stronger constitutive defensive response or are able to maintain a induced defensive response, which in either case permits the plant to compensate for herbivory by allowing growth to resume [18].

Previous studies have indicated that substantial diversity exists within switchgrass (*Panicum virgatum*) populations in terms of plant response to both GB and the yellow sugarcane aphid (*Sipha flava*) [20–22]. However, the molecular mechanisms underlying these responses have yet to be elucidated. Here, global transcriptional responses of switchgrass cultivar Summer plants, an upland tetraploid cultivar adapted to the Upper Midwest of the USA [23], to GB infestation over the course of a 15-day evaluation period were identified using RNA-Seq. This cultivar could serve as a host for GB, and was moderately susceptible in response to GB herbivory, even though GB spent considerable time feeding on leaf phloem, suggestive of a tolerance-response to GB herbivory [20–22]. Unlike other plant-aphid systems, for example, Arabidopsis-green peach aphid [15, 16], corn-corn leaf aphid [24, 25], soybean-soybean aphid [26–28], there is limited information on the underlying physiological responses of switchgrass to insect herbivory, although plant lignin content, peroxidases, and responses to ROS have been implicated in several recent publications [29–31]. The intent of this study was to discover molecular signatures underlying switchgrass responses to GB and contrast these to developmental changes occurring in uninfested control plants over the time course of the experiment.

## Methods

### Plant growth conditions and sample collection

Seeds of cultivar Summer were obtained from field grown plants maintained by the USDA-ARS at their field locations near Mead, NE, USA. Original source of certified cultivar Summer seeds was the Manhattan Plant Materials Center, Manhattan, KS, and subsequently seeds were verified by USDA-ARS scientists based in Lincoln, NE. Fifty switchgrass plants from cultivar Summer were grown in individual Cone-tainers (Ray Leach SC10; Stuewe & Sons, Inc, Tangent, OR) to the L2 stage [32] in a greenhouse under 400-watt high intensity lamps with a 16 h day and 8 h night photoperiod at a temperature of 23° ± 4 °C [21]. The plants were arranged in a 2x3 factorial design consisting of two treatments (infested and control) and three harvest time points: 5-, 10-, and 15- days after infestation (DAI). Ten GB were initially placed on infested plants at day 0. Infested and control plants were individually caged with tubular plastic cages with vents covered with organdy fabric to confine GB on the infested plants. Before leaf samples were taken at each time point, GB were counted and removed and damage rating evaluations were performed on the plants using a 1 to 5 scale [21, 22, 33], where 1 = 10% or less of leaf area with reddish or yellowing discoloration; 2 = 11–30% of leaf area with reddish or yellowing discoloration; 3 = 31–50% of leaf area with reddish or yellowing discoloration; 4 = 51–70% of leaf area with reddish or yellowing discoloration; and 5 = 71% or more of leaf area with severe discoloration, or dead tissue. All leaves present on the plant were collected flash frozen with liquid nitrogen and stored at −80 °C for future processing.

### RNA extraction and sequencing

Four biological replicates (individual plants) were processed from each time point and treatment. A total of 24 RNA samples were isolated from flash frozen leaf samples as previous described in [34, 35] and then purified using RNeasy^TM^ kit according to the manufacturer’s protocols (Qiagen, Valencia, CA) for RNAseq experiments. RNA purity and concentration of the RNA was determined using a Take3 plate and the Direct RNA Quantification Protocol (Bio-Tek, Winooski, VT). Purified RNA quality was validated using the RNA 6000 Nano Kit with the Total RNA Nano Assay for Plants (Agilent, Santa Clara, CA). From the clean RNA samples, 24 TruSeq^TM^ RNAseq libraries utilizing unique indexes were produced according to manufacturer’s protocols (Illumina Inc, San Diego, CA). Individual samples were diluted to a concentration of 10 nM and multiplexed at five samples per lane. Single read 100-bp sequencing was performed on the Illumina HiSeq 2000 system. All RNA-Seq libraries, indexing and sequencing were performed at the DNA Microarray and Sequencing Core Facility at the University of Nebraska Medical Center.

### RNA-Seq analysis

The RNA-Seq datasets analyzed during the current study are available in the SRA repository, Accession number SRP070829; (https://www.ncbi.nlm.nih.gov/sra/?term=SPR070829) and additional data are provided as Additional file 1: Table S1, Additional file 2: Table S2, Additional file 3: Data S1 and Additional file 4: Data S2. Single end 100-bp reads were mapped to the switchgrass genome (v1.1, phytozome.jgi.doe.gov) [36] using Tophat2 [37] with default parameters. Reads with multiple alignments were discarded, and gene expression counts were calculated using the featureCounts function in Subread [38]. Differential gene expression analysis was performed using DESeq2 [39, 40]. Genes differentially expressed (FDR < .05) in at least one of the treatments in the dataset as a whole were identified using the likelihood ratio test, and specific contrasts were tested using the standard multi-factor design workflow with nbinomWaldTest. Heat maps were assembled using z-scores of replicate averages, which is indicative of the number of standard deviations that the expression level of each gene is from the mean expression level of the gene across all treatments. A z-score less than 0 represents a gene expression level less than the mean, while a z-score greater than 0 indicates a gene expression level above the mean.

### Transcriptome mapping statistics

All transcriptomic-related analyses were derived from data shown in Additional file 1: Table S1. An average of 44.4 million 100-bp single-end reads per sample, with a range from (34.9 to 54.6 million reads) were generated from RNA isolated from each sample at 5-, 10-, and 15-DAI (Additional file 1: Table S1), and were mapped to the reference switchgrass genome (version 1.1; www.phytozome.org). There were no significant differences between total reads, mapped reads, or reads mapped to annotated regions between the infested and control samples throughout the time course (Additional file 1: Table S1).

### Gene ontology enrichment analysis

Gene Ontology (GO) enrichment analysis was carried out using GOseq [41]. This R package was designed specifically to analyze GO enrichment in RNA-Seq datasets. Initial GO annotations were taken from the switchgrass genome annotation (v1.1) and expanded with the addition of parental terms. GO categories with fewer than five genes were removed prior to GOseq analysis. Genes were weighted by length and categories with FDR-corrected *p*-values ≤ 0.05 were identified as being enriched with all expressed genes in the data set used as the reference.

### Network analysis

Weighted gene co-expression network analysis (WGCNA) [42, 43] was used to identify gene co-expression modules. Co-expression modules arising from the current study were also compared to modules identified in switchgrass flag leaves [34] to identify any overlapping expression patterns, especially those linked to senescence [44]. Differentially expressed genes (DEGs) were identified using the likelihood ratio test in DESeq2. DEGs were filtered by requiring a FDR ≤ 0.05 and a log_2_ fold change ≥ 2 between the highest and lowest normalized (variance stabilizing transformation) expression values. A signed network was then created from the resulting 18,581 genes with a soft threshold (β) value of 18, a minimum module size of 30, and a merge threshold of 0.25. Cytoscape (version 3.2.0) [45] was used to visualize the resulting network. The topological overlap measure (TOM), calculated by WGCNA, was used as a co-expression measure for pairs of genes. The top four TOMs for each gene, along with the top 0.4% of all TOMs, were used to generate the network, which was drawn in Cytoscape using the AllegroLayout plugin with an edge-weighted Allegro Fruchterman-Reingold algorithm. Edges connecting gene pairs were weighted by their respective TOM values.

### Metabolite and enzyme analysis

A separate experiment was conducted to identify defense metabolites and enzymes that accumulated in GB-infested plants. Five plants each were grown in individual Cone-tainers to the L2 stage and were either infested with GB or maintained as uninfested controls as described for the RNA-Seq experiments. All plants were harvested at 10-DAI, coinciding with peak gene expression observed in the RNA-Seq dataset. Soluble polar metabolites were extracted and derivatized as previously described [46]. Twenty μL aliquots of the extracts were evaporated to dryness under vacuum and derivatized by adding 50 μL of pyridine and 80 μL of N-Methyl-N-trimethylsilyltrifluoroacetamide (MSTFA, #TS-48910 Thermo Scientific, Waltham, MA) and incubated at 60 °C for 2 h. TMS-derivatized samples (1 μL injection) were analyzed on an Agilent 7890B GC with a 5977A MSD with a HP-5MS ultra inert column (30 m x 250 μm x 0.25 μm). GC run conditions consisted of 250 °C inlet and 300 °C MS transfer line, with an initial oven temperature of 60 °C which was increased by 10 °C per minute to 325 °C and maintained for 10 min. Helium was used as a carrier gas with a flow rate of 0.6 mL min^−1^. Putative peak identification and quantitation was performed using Agilent GC-MS MassHunter software. Authentic standards for pipecolic acid (P2519), chlorogenic acid (C3878), and trehalose (T9531) were obtained from Sigma-Aldrich, St.Louis, USA.

ROS as H_2_O_2_ equivalents were measured using Amplex Red Ultra (ThermoFisher, A36006; Waltham, MA) essentially according to [47]. Briefly, 300 μL of 0.1 M sodium phosphate buffer, pH 7.5 was added to 50 ± 2 mg of liquid-nitrogen ground plant tissues. Tissues were subjected to 2 cycles of sonication for 7 s each on a Branson digital sonifier (Model 450; Branson Ultrasonics Corp, Danbury, CT), attached to a microtip and an amplitude setting of 20%. Tubes were placed on ice in between sonification cycles. Samples were vortexed for 30 s following sonification and kept on ice for a further 10 min, prior to centrifugation for 15 min at 14,500 rpm at 4 °C. Triplicate 50 μL aliquots from each sample were used to detect ROS. A standard curve of 0 to 2000 pmoles of H_2_O_2_ was used to calculate ROS equivalents in tissue extracts. Fluorescence of all samples were determined on a Bio-Tek Synergy plate reader (Bio-Tek, Winooski, VT), using an excitation filter of 530 ± 25 nm and an emission filter of 590 ± 35 nm.

Aliquots of 50 ± 2 mg of liquid-nitrogen ground plant tissues obtained from five individual plants (control or infested) harvested 10 DAI were assayed for peroxidase and laccase activities as follows: approximately 10 mg of insoluble polyvinylpolypyrolidone (PVPP, 77627, Sigma-Aldrich, St. Louis, MO) was added to plant samples on ice, followed by the addition of 600 μL of 0.1 M sodium phosphate buffer, pH 7.0 containing 1.6 mM PMSF (P7626, Sigma-Aldrich, St. Louis, MO). Two stainless steel balls (3 mm and 1 mm) were added to each tube, and tubes were placed in prechilled (−20 °C) cryoholders of a 2010 Geno/Grinder (SPEX SamplePrep, Metuchen, NJ). The machine was operated at 1500 rpm for two agitations of 30 s each with an interval of 60 s between shaking. Samples were centrifuged for 15 min at 14,500 rpm at 4 °C. Supernatants were used as a source of enzymes. All assays were performed in a total volume of 200 μL in 96 well microtiter plates at 30 °C. Changes in absorbance were measured using a Bio Tek Synergy HT plate reader (Bio-Tek U.S., Winooski, VT). For peroxidase measurements, samples were diluted 1:5 with 0.1 M phosphate buffer, pH 7.0. For laccase assays undiluted supernatants were used. Peroxidase activity was assayed using 5 μL of diluted extract in a solution containing 20 mM HEPES-NaOH buffer, pH 6.0, 6.75 mM guaiacol and 0.1 M H_2_O_2_ (final concentrations) as described previously [48, 49]. Laccase activity was assayed using 10 μL of undiluted extract in 100 mM sodium acetate buffer, pH 5.0, containing 1.82 mM 2,2′-azino-bis(3-ethybenzothiazoline-6-sulfonic acid) and 10 μg catalase (C-1345, Sigma-Aldrich, St. Louis, MO) in a total volume of 200 μL [50]. Increase in absorbance was followed at 420 nm every 5 min for 30 min at 30 °C. An extinction coefficient of 3.6 x 10^4^ M^−1^ cm^−1^ was used to calculate laccase activities [51].

### cDNA synthesis and real-time qPCR validation

Subsamples of RNA used for RNA-Seq experiments were utilized to generate cDNA libraries for real-time qPCR validation using the Evagreen chemistry on a Fluidigm Biomark HD Instrument (Fluidigm, South San Francisco, CA) using manufacturer supplied protocols (available on-line at https://www.fluidigm.com/). Genes, primers, and amplicon sizes of products are provided in Additional file 2: Table S2. Data obtained from this instrument were analyzed using the Biomark & EP1 software freely available through Fluidigm. Four housekeeping genes were used to generate ΔCt values which were subsequently used for statistical evaluation as described below.

### Statistical analyses

Statistical analyses of GB numbers and plant damage, enzymes and metabolites were performed using ANOVA in Excel. Real-time qPCR data for gene expression analysis were completed according to manufacturer’s protocol (Fluidigm). Relative expression values were statistically analyzed using ANOVA followed by Tukey’s Honestly Significant Difference (HSD) post hoc analysis using p ≤0.05 as a cutoff for significance.

## Results

### GB accumulation and damage ratings

Originally 10 apterous GB were placed on each plant at day zero, and GB numbers continued to increase over the duration of the experiment. The highest GB numbers were observed at 15 days after infestation (DAI) (Table 1). Similarly, plant damage ratings increased with time and were significantly higher at 15-DAI. The average damage rating of 2.4 ± 0.2 at 15-DAI indicated that at least moderate damage had occurred to infested plants. No GB (or other insects) were found on the control plants.

**Table 1:** Aphid numbers, damage ratings and leaf stage of samples collected throughout the time course. (*n* = 4 samples per treatment)

Sample	Total Aphid Number	Damage Rating	Leaf Stage
Day 5 Infested	25 ± 16.8^a^	1.4 ± 0.4	L3
Day 5 Control	0	1	L3
Day 10 Infested	33.8 ± 13.8^a^	1.9 ± 0.2^a^	L4
Day 10 Control	0	1	L4
Day 15 Infested	57 ± 27.1^a^	2.4 ± 0.2^a^	L4
Day 15 Control	0	1	L4

Means and standard errors are shown; samples with letter “a” are statistically different (*p*-value ≤ 0.05) from the control sample at that specific time point

### GB-infestation significantly alters transcriptomes in switchgrass plants

**Fig. 1:**
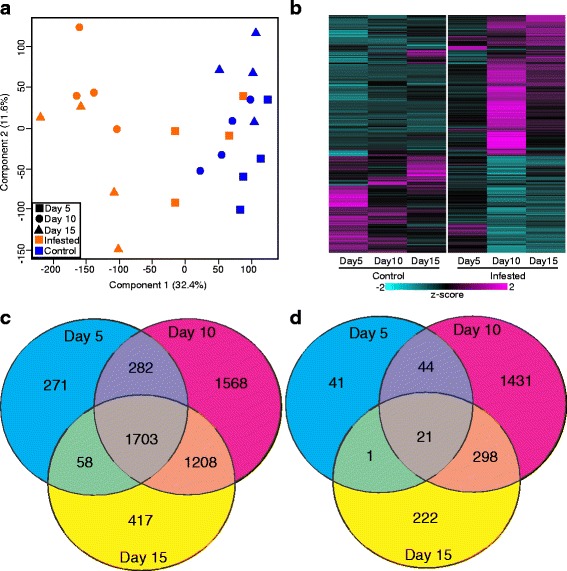
Overview of transcriptomic data. **a** PCA of transcriptomic data, *Blue* symbols, controls; *Orange* symbols, GB-infested plants; *squares* 5-DAI; *circles*, 10-DAI; and *triangles*, 15-DAI harvest dates. **b** Heatmap of global changes in differentially expressed genes based on z-scores where *cyan* is low expression and *magenta* is high expression. *Left* panel controls, and *right* panel GB-infested plants. **c** Venn diagram of genes induced by infestation relative to control of same time point. **d** Venn diagram of genes suppressed by GB infestation relative to control of same time point. Numbers within each region indicate common and unique genes within each sector

Principal component analysis (PCA) effectively separated the different transcriptome samples by treatment and partially separated the samples by time point within each treatment (Fig. 1a), which suggests changes in transcriptional profiles over the 15 day time course arose from both development-related changes and stress conditions associated with GB infestation. By 5-DAI, the transcriptomes of the infested plants were partially differentiated from the control plants by the first principal component (PC1, which accounted for 32.4% of the variance), and by 10-DAI, the transcriptomes from uninfested plants were fully differentiated from the transcriptomes of the infested plants along the PC1 axis (circles, Fig. 1a). By 15-DAI, transcriptomes of the uninfested plants were partially differentiated from the transcriptomes of 10-day uninfested plants along the PC2 axis (11.2% of the variance), which could be linked to developmental changes.

### Identification of differentially expressed genes during GB infestation

Global changes in differentially expressed genes (DEGs) were identified using an FDR ≤ 0.05 and a fold change of ≥ 2 (Fig. 1b), and in addition qPCR was performed on a select list of the genes identified as differentially expressed to corroborate the findings of the RNA-Seq experiment (Fig. 7). In control plants, 762 and 779 genes were differentially expressed at 5-DAI, relative to 10- and 15-DAI respectively (Fig. 1b), which is likely due to the developmental changes of the plants through the time course. In infested plants, the maximum number of DEGs occurred at 10-DAI (6558), when compared against uninfested controls, consistent with the PCA data. However smaller numbers of DEGs were also observed at 5 (2425) and 15-DAI (3931; Fig. 1b).

A total of 7565 unique genes were identified as being differentially expressed due to GB infestation when comparing each infested time point to the corresponding uninfested controls. A majority of these DEGs were upregulated in response to GB feeding (5507 genes) compared to the 2058 genes that were downregulated. Among the upregulated DEGs, 271; 1568; and 417 were exclusively upregulated at 5, 10, and 15-DAI respectively, while 1703 were consistently upregulated and 21 were consistently downregulated across all time points (Fig. 1c, d). More DEGs were shared between the 10- and 15-DAI plants compared to the other time points (Fig. 1c). The maximum number of downregulated DEGs (1431) was observed at 10-DAI, and far fewer repressed DEGs were observed at 5 (41) and 15-DAI (222; Fig. 1d).

### GB infestation activates cellular oxidative responsive pathways and suppresses photosynthesis-related pathways

Four different gene ontology (GO) enrichment comparisons were performed to identify up/downregulated GO biological processes in: (1) DEGs in common across all time points in the aphid-infested plants; (2) DEGs at 5 DAI; (3) DEGs at 10-DAI; and (4) DEGs at 15-DAI (Additional file 3: Data S1).

The 55 GO biological process terms enriched in upregulated genes common to all three time points in GB-infested plants included: oxidation-reduction process (GO:0055114), response to biotic stimulus (GO:0009607), and defense response (GO:0006952). Eighteen biological process GO terms were enriched in infested plants 5-DAI (271 DEGs; Fig. 1c) including gene expression (GO:0010467) and cellular biosynthetic processes (GO:0044249; Additional file 3: Data S1). By 10-DAI, the seven enriched GO terms associated with upregulated genes in infested plants included transmembrane transport (GO:0055085), single-organism process (GO:0044699), and oxidation-reduction process (GO:0055114). In contrast, the nine significantly enriched terms among the 417 upregulated DEGs that were specific to the 15-DAI plants (Fig. 1c) were associated with protein phosphorylation (GO:0006468) (Additional file 3: Data S1).

No GO terms were significantly enriched in genes downregulated at all three time points or 5-DAI in infested plants (21 and 41 genes respectively, Fig. 1d). The 1431 DEGs downregulated after 10-DAI in GB-infested plants were enriched in 24 biological process terms including biological regulation (GO:0065007), peptide transport (GO:0015833), and nitrogen compound transport (GO:0071705) (Additional file 3: Data S1). Cell redox homeostatic processes (GO:0045454) and photosynthesis (GO:0015979) were among the four enriched in DEGs downregulated in infested plants at 15-DAI.

### Genes associated with chlorophyll, carbon, and nitrogen metabolism are significantly affected by GB herbivory

**Fig. 2:**
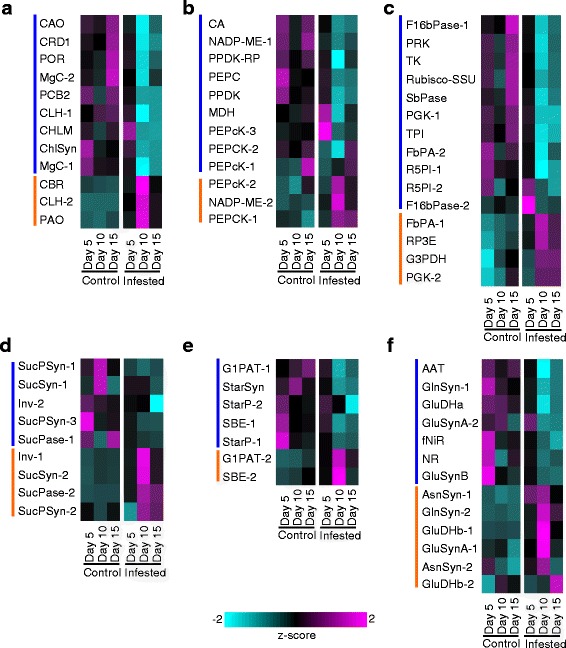
Genes differentially expressed in control and GB-infested (infested) plants associated with primary plant metabolism. *Blue* bars denote genes upregulated in control plants and *orange* bars denote genes upregulated in infested plants. In all cases, differential expression of genes is based on z-scores where *cyan* is low expression and *magenta* is high expression (**a**) Chlorophyll biosynthesis and degradation. **b** Photosynthesis. **c** Calvin Cycle. **d** Sucrose metabolism. **e** Starch metabolism. **f** Nitrogen metabolism. Gene abbreviations, identities, and normalized transcript counts are provided in Additional file 4: Data S2

**Fig. 3:**
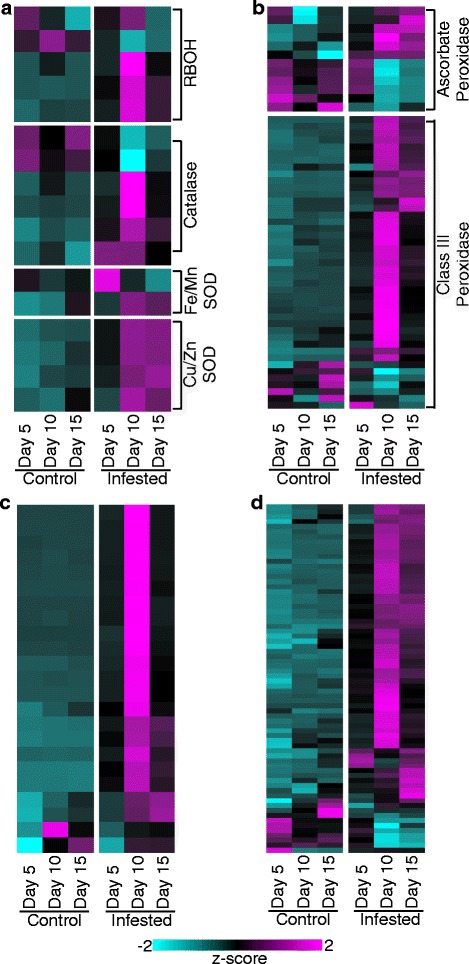
Genes differentially expressed in control and GB-infested (infested) plants associated with plant redox metabolism. **a** RBOHs, Catalases, and SODs. **b** Ascorbate peroxidases, and class III secreted peroxidases. **c** Laccases. **d** GSTs. Other details as described for Fig. 2

**Fig. 4:**
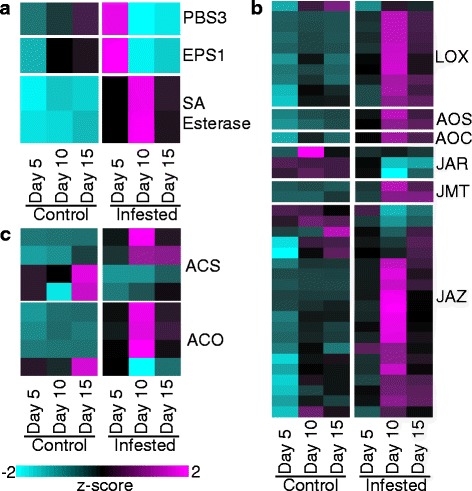
Genes differentially expressed in control and GB-infested (infested) plants associated with three phytohormone metabolic pathways. **a** Salicylic acid metabolism. **b** Jasmonic acid metabolism. **c** Ethylene metabolism. Other details as described for Fig. 2

**Fig. 5:**
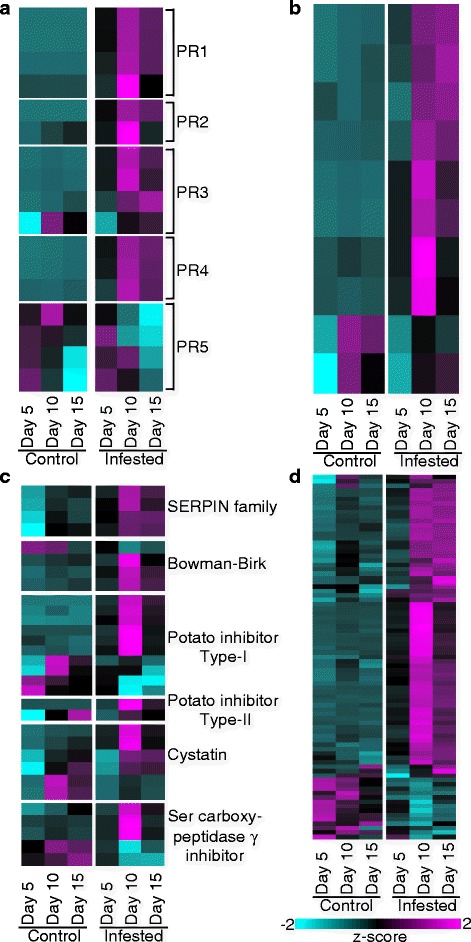
Genes differentially expressed in control and GB-infested (infested) plants associated with plant defense. **a** PR genes. **b** Chitin-related genes. **c** Protease Inhibitors. **d** NB-LRRs. Other details as described for Fig. 2

**Fig. 6:**
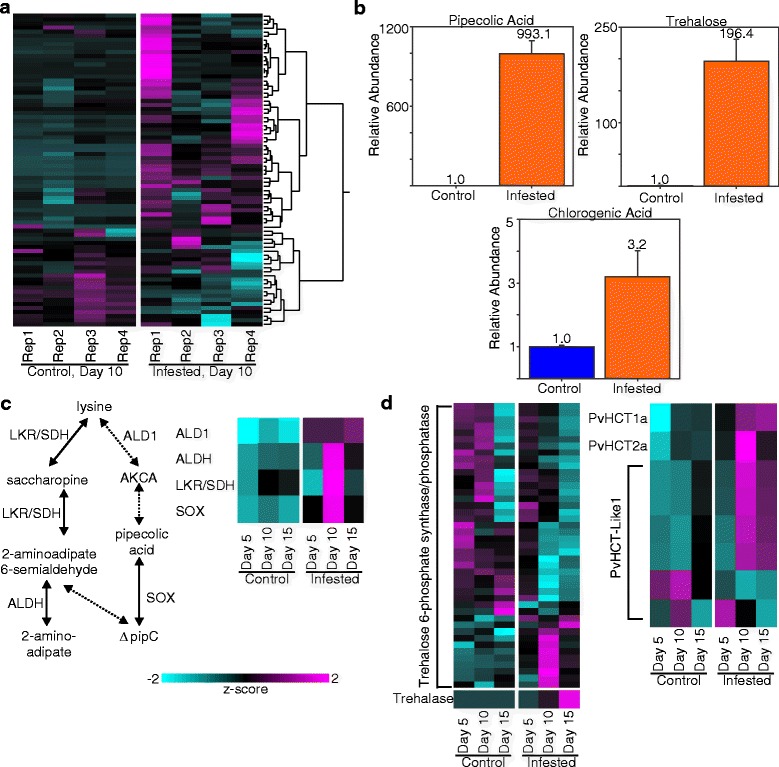
Defense-related metabolite levels and transcript abundances for associated biosynthetic pathways in control and GB-infested (infested) plants. **a** Differential metabolite levels in four biological replicates (Rep1-4) from control and infested switchgrass plants 10-DAI analyzed by GCMS. **b** Specific metabolite levels of pipecolic acid, trehalose and chlorogenic acid in control and GB-infested plants at 10-DAI, validated using authentic standards. **c** Metabolic pathway for biosynthesis of pipecolic acid (ΔpipC), adapted from Zeier, 2013, *left* panel, and transcript abundances (*right* panel). **d** Transcript abundances of genes associated with trehalose metabolism (*left* panel) and chlorogenic acid (*right* panel). Other details as described for Fig. 2

**Fig. 7:**
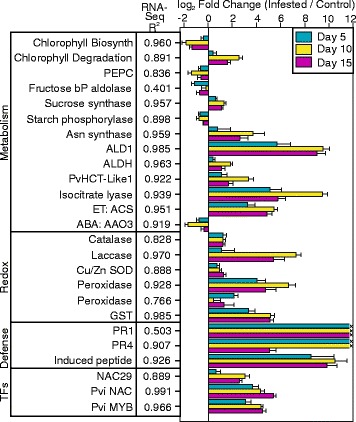
Real-time qPCR validation of gene expression. Expression levels of select genes up/down regulated in the RNA-Seq datasets were analyzed by real-time qPCR. The input RNA used for RNA-Seq studies were used as the source material for qPCR analyses. Gene annotation is provided in the first column and separated by functional classes: metabolism, redox, defense and transcription factors (TFs). The second column lists the correlation coefficients for individual gene expression between RNA-Seq and qPCR analyses. The Log_2_-fold change (infested/control) for genes on 5 (*cyan*), 10 (*yellow*), and 15 DAI (*magenta*) respectively are shown. Gene ids, primers, and amplicon sizes are provided in Additional file 2: Table S2

**Fig. 8:**
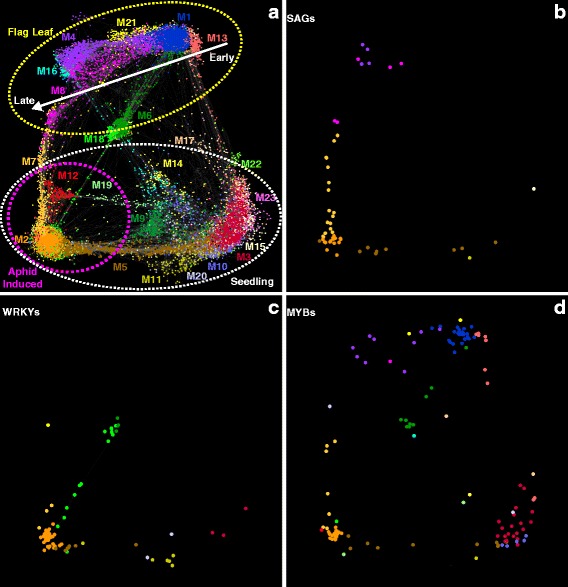
Gene co-expression networks and module assignment for a combined flag leaf development [34], and current RNA-Seq datasets. **a** 23 modules were detected between 17,637 genes (nodes). The top half of the network (*yellow circle*) consists of genes primarily expressed in flag leaves (early development on the right, senescence onset on the left) *white arrow*. Bottom *white circle* of the network consists of genes primarily expressed in seedlings used in the current study (GB-induced host genes in the lower-left; *magenta circle*). Module 2 (M2, *orange dots*) contained the network of co-expressed genes associated with GB infestation. Module 7, (M7, *yellow dots*) contained genes co-expressed in GB-infested plants and during senescence of flag leaves. Module 18 (M18, *green dots*) consisted of genes co-expressed in expanding flag leaves and during the latter stages of GB-infestation. Module profiles within this network are shown in supporting Additional file 6: Figure S2. **b** SAGs associated with different modules; dots color coded as in Panel **a**. **c** WRKY transcription factors; dots color coded as in Panel **a**. **d** MYB transcription factors; dots color coded as in Panel **a**. Gene abbreviations, identities, and normalized transcript counts are provided in Additional file 4: Data S2

**Fig. 9:**
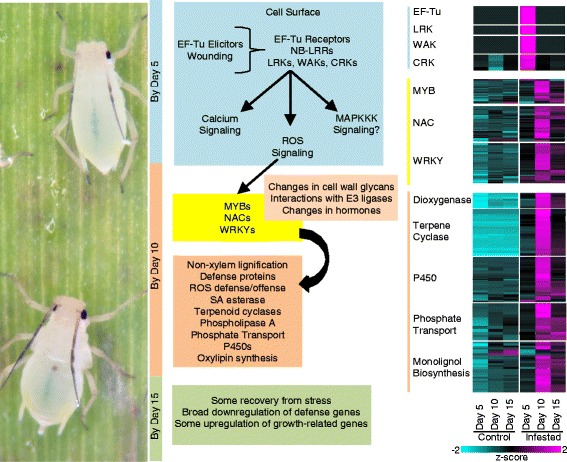
Upland tetraploid switchgrass responses to GBs are robust and include a plethora of pathways. *Left* panel, GBs feeding on switchgrass leaves. Center panel summarizes transcriptional and some metabolite evidence for predicted changes occurring in GB-infested plants within the time course of the experiment. *Light blue* bar and box, changes occurring within 5-DAI; *Light ochre* bar and boxes, changes occurring within 10-DAI; *Yellow* box highlights some of the key transcription families that are part of the DEGs. *Light green* bar and box, changes in transcriptomes that support recovery from GB-induced stress and recovery occurring by 15-DAI. *Right* panel, heatmap of DEGs associated with each harvest date. Colors of bars are as described for center panel. Other details as described for Fig. 2

Expression levels (average normalized mapped reads) for all of the genes described in Figs. 2, 3, 4, 5, 6, 7, 8 and 9 are provided in Additional file 4: Data S2. In control plants, expression of chlorophyll biosynthetic genes generally increased over the 15-day time course (Fig. 2a, blue bar), with minimal changes occurring in expression levels for chlorophyll degradative genes (Fig. 2a, orange bar). In contrast, genes with roles in chlorophyll biosynthesis were significantly downregulated by 10- and 15-DAI in the GB-infested plants, and chlorophyll catabolic genes, namely chlorophyll(ide) b reductase (*CBR*), chlorophyllase 2 (*CHL2*), and pheophorbide A oxidase (*PAO*) were induced by 5-DAI (1.2 to 2 fold) and upregulated (2.8 to 4 fold) by 10-DAI (Fig. 2a; Additional file 4: Data S2) in infested plants. Although expression levels of genes involved in chlorophyll catabolism were reduced 15-DAI in comparison to 10-DAI in infested plants (Fig. 2a, orange bar), they were still significantly greater than those observed for the 15-DAI uninfested control plants (Fig. 2a, blue bar). Consistent with an upregulation in chlorophyll catabolism and reduced chlorophyll biosynthesis in GB-infested plants, chlorophyll content in GB-infested plants 10-DAI were significantly lower than in comparable uninfested control plants (Additional file 5: Figure S1a).

Changes in chlorophyll metabolism were mirrored by downregulation of the majority of genes involved in carbon fixation (Fig. 2b) and the Calvin cycle (Fig. 2c) in infested plants by 10-DAI, although there appeared to be a recovery in transcript levels for several of these genes by 15-DAI (Fig. 2b, c). However, transcript levels for a few genes including PEPcarboxylase kinase (*PEPcK-2*), NADP-Malic enzyme (*NADP-ME-2*), and phosphoenolpyruvate carboxykinase (*PEPCK-1*) were upregulated in the 10-DAI plants (Fig. 2b, orange bar). Additionally, cytosolic, but not chloroplastic, fructose bisphosphate aldolase (*FbPA-1*), ribulose phosphate 3 epimerase (*RP3E*), glyceraldehyde 3-phosphate dehydrogenase (*G3PDH*), and phosphoglycerate kinase (*PGK-2*) associated with the Calvin cycle were also consistently elevated in infested plants over the time course (Fig. 2c, orange bar).

Transcriptional profiles of genes involved in sucrose synthesis and degradation are shown in Fig. 2d. Three sucrose-phosphate synthase genes (*SucPSyn*) were expressed, two of which were more highly expressed in control plants compared to the infested plants. In contrast, a pair of sucrose synthase genes (*SucSyn-2*) was more highly expressed at all three time points in the GB-infested plants compared to control plants. A concomitant increase in invertase (*Inv1*; Fig. 2d) transcript levels was observed in the infested plants. Conceivably, these transcriptional changes signal increase sucrose breakdown and decreased sucrose production due to reduced photosynthetic capacity of the infested plants (see Fig. 2b, c). In concert, starch synthesis was also apparently affected by GB herbivory (Fig. 2e). A major glucose-1-phosphate adenylyltransferase gene (*G1PAT-1*; Fig. 2e, orange bar) and several other starch biosynthesis genes were significantly downregulated in the infested plants compared to the control plants. Downregulation of the starch-biosynthesis transcripts had occurred as early as 5-DAI (Fig. 2e). It is conceivable that some of the contrasting patterns in the expression of the genes associated with sucrose metabolism were under plant developmental control (higher in uninfested plants) and some responded to GB herbivory.

Nitrogen metabolism was also significantly impacted by GB herbivory (Fig. 2f). Transcripts for nitrate reductase (*NR*) and ferredoxin-dependent nitrite reductase (*fNiR*) were downregulated by 5-DAI in the GB-infested plants, and remained depressed even 15-DAI (Fig. 2f). Genes required for assimilating ammonia into amino acids in the chloroplast were also downregulated in GB-infested plants, including glutamine synthetase 1 (*GlnSyn-1*), ferredoxin-dependent glutamine-2-oxoglutarate amino transferase (*GluSynA-2*), and NADP + −dependent glutamate dehydrogenase (*GluDHa*) (Fig. 2f, blue bar). Conversely, cytosolic glutamine synthetase (*GlnSyn-2*), asparagine synthetases (*AsnSyn-1* and *AsnSyn-2*), and NAD + −dependent glutamate dehydrogenase (*GluDHb-1 and GluDHb-2*) were upregulated in infested plants, generally with maximum expression levels observed 10-DAI (Fig. 2f, orange bar), suggestive of increased protein turnover. Together these data signal a potential decline in N-assimilation within chloroplasts, consistent with the apparent slowdown in photosynthesis described earlier.

### Infested plants upregulate genes related to ROS signaling and mitigation

Expression profiles of gene families involved in ROS metabolism are shown in Fig. 3. Expression of five reactive-burst oxidase (RBOH) genes was detected in both infested and uninfested plants. Of these, only one gene was upregulated in the control plants while three RBOHs were substantially elevated by 10-DAI in infested plants (Fig. 3a). Similarly, two catalases were upregulated in control plants, but four were significantly induced within 10-DAI in the infested plants. Only one Fe/Mn superoxide dismutase (SOD) gene was strongly upregulated in infested plants 5-DAI, and this same gene was downregulated in plants at 10- and 15-DAI. In contrast, a second Fe/Mn SOD was upregulated at 10-DAI, and its expression level was sustained through 15-DAI in infested plants. However, transcripts for this specific Fe/Mn SOD were also detected in high abundances in 15-DAI uninfested plants, suggesting that elevated expression of this gene might be linked to developmental processes, and possibly in the recovery of plant growth in the infested plants (Fig. 3a). Four Cu/Zn SOD genes were significantly upregulated in GB-infested plants by 10-DAI and transcript counts remained elevated in plants 15-DAI, indicating these SODs might be strong candidates for detoxification of excess ROS produced as a defensive response to GB. Supporting these findings, cellular ROS (as H_2_O_2_ equivalents) was significantly greater in GB-infested plants 10-DAI (Additional file 5: Figure S1b).

A total of 12 switchgrass ascorbate peroxidase genes were differentially expressed (Fig. 3b). Six of these genes were expressed at higher levels in control plants in at least two time points and six were significantly upregulated in the infested plants with maximal expression levels occurring at later time points (10- and/or 15-DAI) (Fig. 3b). Four of the six genes upregulated in the infested plants encoded ascorbate peroxidases destined for the cytoplasm, and conversely four of the six genes upregulated in control plants were targeted to plastids (Additional file 4: Data S2).

Forty-three class III peroxidase genes were differentially expressed (Fig. 3b). A majority of these genes were upregulated in response to GB feeding as early as 5-DAI (black coloring in 5-DAI compared to cyan coloring in control plants), with peak expression generally occurring 10-DAI (Fig. 3b), although three peroxidase genes were upregulated in infested plants 15-DAI. In contrast, transcripts for seven other peroxidases were expressed more highly in uninfested plants, suggesting an involvement in developmental processes (Fig. 3b). Recently, peroxidases present in a syntenic region of the genomes of switchgrass, sorghum (*Sorghum bicolor*), and foxtail millet (*Setaria italica*) have been shown to be variably responsive to GB feeding [29], supportive of the findings reported here.

Increases and decreases in gene expression were observed for a large number of laccases (Fig. 3c) and glutathione-S-transferases (GSTs; Fig. 3d) as responses to GB herbivory.

Supporting the role of peroxidases and laccases in response to GB-herbivory, both total peroxidase (using guaiacol as a substrate) and laccase activities were significantly elevated in GB-infested plants 10-DAI (Additional file 5: Figure S1c, S1d).

### Genes associated with JA, SA, and ET biosynthesis are upregulated during infestation

Phytohormone metabolic pathways are commonly used by plants for defense against both pests and pathogens. Expression levels of four genes associated with salicylic acid (SA) metabolism were significantly altered during GB infestation, including a hydroxybenzoate-glutamate ligase (*PBS3*), a BAHD acyl transferase (*EPS1*),and two SA esterases (Fig. 4a). The SA esterases were significantly induced during infestation and low transcript levels were observed in the control samples. A greater effect on genes involved in jasmonic acid (JA) metabolism was observed with 38 total DEGs identified across the time course (Fig. 4b). These genes included 10 lipoxygenase (*LOX*), two allene oxide synthase (*AOS*), and one allene oxide cyclase (*AOC*) genes, all of which were induced by GB-infestation (Fig. 4b). In contrast, three jasmonic acid-amido synthetases (*JAR*) were downregulated in infested plants (Fig. 4b). There were also twenty jasmonate-ZIM domain (*JAZ*) genes (Fig. 4b) that had variable expression levels, with the majority being upregulated during infestation but some also being downregulated during the two later time points, which suggests that the expression of *JAZ* genes may be fine-tuned during GB defense responses. In addition to SA and JA associated DEGs, DEGs associated with the ethylene (ET) metabolism were also observed. These included four acetyl-CoA synthetase (*ACS*) and four ACC oxidase (*ACO*) genes, most of which showed the greatest upregulation at 10-DAI (Fig. 4c).

### Switchgrass plants mount a significant defensive response against GBs

The incursion of pests and pathogens can elicit the upregulation of a number of plant gene families associated with defense, including pathogenesis responsive (*PR*) genes, chitinases, proteases, inhibitors of insect digestive enzymes, and any of a number of resistance gene homologs (*RGHs*) belonging to the NB-LRR family of proteins [24, 52]. In Summer switchgrass plants challenged with GB, there was a dramatic upregulation in all of the classes of defensive genes including *PR1* through *PR4*, but not *PR5* families (Fig. 5a).

Transcripts for genes containing chitinase (PF00182) and chitin recognition (PF00187) domains induced by GB (Fig. 5b) typically displayed maximum expression levels that were five- to ten- fold higher than those expressed in control non-infested plants (Additional file 4: Data S2). Several of these genes were significantly induced within 5-DAI, although two chitinases (*Pavir.Aa01411* and *Pavir.Ab02160*) had expression profiles indicative of developmental or age-related regulation (Fig. 5b bottom) and were upregulated at later time points (10- or 15-DAI in control plants).

Six major families of proteinase inhibitors (PIs) were induced in response to GB feeding that included members of the SERPIN, Bowman-Birk, potato inhibitor type-I and type-II, cystatins, and serine carboxypeptidase-γ-inhibitors families (Fig. 5c). Maximal expression was generally observed 10-DAI, although in many cases their induction was significantly elevated 5-DAI (black coloring in 5-DAI compared to cyan coloring in control plants; Additional file 4: Data S2). None of these PI families appeared to have exclusive roles in GB defense, because at least one gene from every PI class was more highly expressed in uninfested plants (Fig. 5c).

Profiles for the differentially expressed NB-LRRs (PF00931) are shown in Fig. 5d. Evidence for both age-related and GB-inducible changes in NB-LRR gene expression was observed. In the infested plants, two different expression profiles were observed: (1) several NB-LRR genes were strongly upregulated 10-DAI with decreased expression levels observed 15-DAI in infested plants, and (2) other NB-LRRs were induced strongly 10-DAI and maintained high expression through 15-DAI in infested plants (Fig. 5d).

### GB elicit changes in defense metabolites in infested switchgrass tissues

A total of 70 features with putative identification quality scores >70 were detected in the metabolite analysis. Despite some slight variation observed among the metabolomes of the biological replicates of the control and infested plants, it was evident that the metabolomes had been significantly affected by GB herbivory (Fig. 6a). Three GCMS peaks were substantially enriched in GB-infested plants, and yielded mass spectra attributable to pipecolic acid, chlorogenic acid, and trehalose. All three compounds have been implicated in plant defense [53–55] and were therefore validated using authentic standards. Pipecolic acid was found at low levels in control plants but was elevated almost 1000-fold in the infested plants. Trehalose was enriched by almost 200-fold in the infested plants and chlorogenic acid was enriched about 3-fold in the infested plants (Fig. 6b). An analysis of genes involved in these three pathways was performed next.

The predicted pathway for the formation of pipecolic acid from lysine is shown in Fig. 5c (adapted from [56]). The switchgrass genes encoding LL-diaminopimelate aminotransferase/aldehyde dehydrogenase (*ALD1*) and sarcosine (pipecolic acid) oxidase (*SOX*) were significantly upregulated in the infested plants by approximately 100-fold relative to control plants by 5-DAI, and the elevated expression levels for these genes were sustained through 15-DAI (Additional file 4: Data S2). Additionally, two other switchgrass lysine degradation pathway genes, namely lysine ketoglutarate reductase/saccharophine dehydrogenase (*LKR/SDH*) and L-aminoadipate semialdehyde dehydrogenase (*ALDH*), were strongly upregulated by 10-DAI and retained higher expression than control plants at 15-DAI (Fig. 6c; Additional file 4: Data S2).

A large number of trehalose-6-phosphate synthase and trehalose-6-phosphate phosphatase genes were found in the switchgrass genome, but given their structural similarities and lack of biochemical characterization, it was not possible to clearly discriminate between genes coding for either enzyme using their amino acid sequences alone. However, when considered as a group, significant expression changes were documented both in control and infested plants (Fig. 6d). One set of trehalose-6-phosphate synthase/phosphatase genes was significantly upregulated in GB-infested plants 10-DAI. In contrast, there appeared to be a greater diversity in the expression profiles of these genes in the control plants across all the three harvest dates (Fig. 6d). Transcripts were mapped to two trehalases, both of which were significantly upregulated in infested plants by 15-DAI, whereas transcript levels for these two genes were relatively unchanged in control plants (Fig. 6d, Additional file 4: Data S2).

Switchgrass plants also produce chlorogenic acid, through the action of specific hydroxycinnamoyl CoA-shikimate/quinate hydoxycinnamoyl transferases (HCTs [55]). *HCT1a* and *HCT2a* genes were upregulated in GB-infested plants as early as 5-DAI with substantial increases in transcripts by 10-DAI (Fig. 6d). Of the six expressed *HCT-Like1* genes, four were significantly upregulated by 10-DAI in infested plants, although the expression levels for these four genes also increased over the time course in the control plants (Fig. 6d). Two *HCT-Like1* genes were expressed at higher levels in the control plants relative to the infested plants (Fig. 6d).

### Real-time qPCR validates gene expression profiles

Twenty five genes representing key pathways, including chlorophyll biosynthesis and degradation, C and N-metabolism, hormones, redox, plant defense and three specific transcription factors, were selected for validation by real-time qPCR using the identical input RNA that was used for RNA-Seq studies (Fig. 7). Four housekeeping genes (Additional file 2: Table S2) were used to calculate ΔCt and log_2_-fold change (infested/controls). Real-time qPCR in general had correlation coefficients of > 0.7, and in many case >0.9, with the RNA-Seq expression values for most genes queried (Fig. 7). *Fructose bis-phosphate aldolase* (R^2^ = 0.401) and *PR1* (R^2^ = 0.503) were the two exceptions. In the case of *PR1*, little if any amplification occurred in control samples. Real-time qPCR essentially validated RNA-Seq results.

### Network analysis identifies gene sets associated with leaf senescence, and recovery of leaf function in response to GB herbivory

Because RNA-Seq data indicated that significant changes in leaf metabolic pathways associated with senescence occurred in GB-infested switchgrass, a comparison between gene networks associated with flag leaf development and senescence [34] and GB herbivory (this study) was performed to discover networks unique and common to these two processes.

A total of 23 co-expression modules (M1 through M23) among 17,637 genes were detected in the combined flag leaf and GB herbivory datasets (Fig. 8a, Additional file 6: Figure S2). In general, the networks of genes correlating with flag leaf development (top yellow dotted circle) were separable from the networks found in the GB-infested and uninfested seedlings (Fig. 8a, bottom white dotted circle). Additionally, strong gradients were present in networks associated with flag leaf development (early to late stages, white arrow, flag leaves) and response to GB feeding over the 15-day time course (magenta dotted circle; Fig. 8a, lower left corner) that suggests several of the gene expression networks were exclusively associated with flag leaf development (modules 1, 4, and 21), GB-infestation (modules 2, 7, 12, and 18), or developmental events during early plant growth, (modules 11, 15, 20, and 22) and with flag leaf senescence (modules 7, 8, 12).

Sixty-five putative switchgrass orthologs to senescence-associated genes (SAGs) were identified in flag leaves [34], of which 54 were within the combined flag leaf development and GB feeding datasets (Fig. 8b; Additional file 4: Data S2). Most of these SAGs were found in modules 2 (orange dots), 7 (yellow dots), and 8 (magenta dots) (Fig. 8b). Expression patterns of SAGs in GB-infested plants showed evidence of a limited progression towards senescence as only a subset of the SAGs were induced. For example, module 2 contained 18 SAGs induced by GB infestation. Among these genes were three homologs to Arabidopsis SAG2 (*AT5G60360*, coding for an aleurain-like thiol protease; [57]), three catalase genes including the *CATALASE 2* ortholog [34], and one homolog to Arabidopsis *ANAC029*, a known regulator of leaf senescence [58]. It is plausible that triggering some components of senescence pathways leads to reprogramming of leaf metabolism away from nutrient assimilation and towards the biosynthesis of defense molecules. Module 7 contained 16 SAGs which were expressed at similar levels during both GB-induced stress and flag leaf senescence. The majority of these SAGs included homologs to ANAC029 and genes associated with chlorophyll degradation, which all have established roles during senescence [34, 59, 60]. Module 8 contained five SAGs (Additional file 4: Data S2) which were only expressed during flag leaf senescence [34] and not induced in the GB-infested plants, supportive of a limited progression toward senescence under insect pressure.

A total of 83 *WRKY* genes out of 250 *WRKYs* identified in the switchgrass genome [44] were found in the combined network (Fig. 8c). WRKYs are key regulators of plant biotic and abiotic stress responses [61], and possible roles for WRKYs in switchgrass flag leaf senescence have been proposed [44]. Forty-two WRKYs were present in module 2 (Additional file 4: Data S2; GB-infested). Eighteen of these 42 WRKYs were also associated with flag leaf senescence (see Fig. 5 in [44]). Similarly, of the 14 WRKYs assigned to module 18 (Fig. 8c, Additional file 4: Data S2), thirteen were also present in the transcriptomes of expanding flag leaves (see Fig. 5 in [44]). These data provide evidence for both the overlap and divergence between senescence and defense response to GB herbivory. As examples, *PviWRKY29* and *PviWRKY117*, found in module 2, are orthologous to Arabidopsis WRKYs implicated in leaf senescence, biotic stress response, and P nutrition [44, 62, 63]. *PviWRKY54*, found in module 7 (Additional file 4: Data S2), is an apparent ortholog to *ATWRKY28*, which influences plant responses to stress by modulating SA biosynthesis [64]. Within module 18, *PviWRKY175* is orthologous to *ATWRKY33* which encodes a transcriptional regulator modulating responses to fungal infection by directly regulating genes involved in SA, JA, and ET signaling and cross-talk [65]. However, the actual roles of these specific PviWRKYs in plant defense and growth processes in switchgrass remain to be determined.

Network analysis of MYBs detected expression patterns associated with flag leaf development or GB feeding for 136 MYB genes (Fig. 8d, Additional file 4: Data S2). In contrast to the distribution of the WRKY genes (Fig. 8c), the MYBs were represented across the entire network, highlighting their broad roles in regulating plant processes, including secondary cell wall formation [66, 67]. Plant cell wall fortification appears to be a strong response of switchgrass plants to GB herbivory, and specific MYBs could be regulating these processes. Module 2 contained 27 MYBs (Additional file 4: Data S2), and two of these MYBs (*Pavir.Aa01159 and Pavir.J10932*) encode close homologs to sorghum *SbMyb60* (*Sobic.004G273800*). Constitutive overexpression of *SbMyb60* in sorghum plants resulted in higher expression levels of monolignol biosynthesis genes as well as ectopic lignin deposition around the midrib and vascular bundles in leaves [29]. Moreover, module 2 was enriched with several genes encoding lignin biosynthetic enzymes (Additional file 4: Data S2), which suggests switchgrass homologs of *SbMyb60* might also be linked to monolignol biosynthesis to stimulate cell wall fortification. Module 7 contained seven MYBs, whose Arabidopsis orthologs have largely undescribed roles in plant physiology [68, 69]. In contrast, module 18 contained only one MYB, *Pavir.J12840* (Fig. 8d, Additional file 4: Data S2), an apparent ortholog to ATMYB12. ATMYB12 regulates a number of different pathways, including the synthesis of flavonols that provide insect-resistance [70]. Possibly, the switchgrass ortholog could be influencing production of similar defensive compounds. Overall this network analysis provided a detailed map of the transcriptional changes resulting from GB infestation and highlighted expression profiles of key transcription factors that could underlie the defensive responses of switchgrass to GB.

## Discussion

### Switchgrass transcriptomes are significantly modulated by GB feeding

As early as 5-DAI, significant differences in the transcriptomes obtained from GB-infested and uninfested plants were already apparent. Among the early signs of defense responses were changes in C and N metabolism in the infested plants. Suppression of photosynthesis and diversion of carbon appear to be universal early responses in plants to aphid feeding [2, 16, 49, 71], and in this regard, switchgrass responses to GB appears to be similar. Other molecular signatures in infested plants 5-DAI were suggestive of the involvement of both mechanically–triggered and elicitor-triggered plant responses. In the former class were a number of wall-associated kinases including, *Pavir.Ab01425; Pavir.Ib00075; Pavir.J04391*, whose encoded proteins were orthologous to FERONIA (FER) and HERCULES RECEPTOR KINASE 1 (HERK-1). Both FER and HERK-1 transduce mechanical signals in Arabidopsis and function by modulating calcium fluxes, leading to subsequent downstream effects [72]. Two putative switchgrass orthologs to the Arabidopsis elongation factor Tu (EF-Tu) receptor (*Pavir.J26110 and Pavir.J27646*) were significantly upregulated only in infested plants 5-DAI. EF-Tu receptors are known components of the microbe-associated molecular pattern (MAMP) recognition in plants [73, 74] and recognize EF-Tu secreted by bacteria. Aphid honeydew also contains EF-Tu protein and has been suggested to be involved in plant-aphid interactions [75]. Our data would support this hypothesis. However, confirmation of this specific interaction at a biochemical level has yet to be performed.

Other early markers of the response to aphid feeding were specific upregulation of genes encoding wall-associated kinases and the defense-related NB-ARC domain (NB-LRR) proteins (*Pavir.J15505; Pavir.J19633*). In other plants, NB-LRRs have been identified as resistance genes (*R* genes) for specific insects [17, 24]. Elevated expression for genes related to cell wall structure and cytoskeleton, specifically in GB-infested plants harvested at 5-DAI (*Pavir.Ib01358*, *Pavir.Ia03598*, *Trichome birefringence like*, *TBL*; *Pavir.Aa00167*, Myosin XI), might be linked to the signaling cellular changes accompanying activation of the many wall related sensor kinases. In Arabidopsis, the orthologs to the switchgrass TBLs impact the formation of crystalline cellulose in the secondary cell walls through interaction with cellulose synthases [76] and myosin XI participates in the movement of cellular organelles [77], nuclear shape, and plant posture [78]. Myosin IX aids in the formation of effective barriers against pathogens by directing the trafficking of materials needed for cell wall fortification [79], suggestive for a similar role for Myosin IX in switchgrass responses to GBs.

### ROS signaling and mitigation are a component of switchgrass response to GB herbivory

ROS are a well-established component of plant response to insect herbivory [16, 80, 81]. Indeed, the ability of a plant to effectively scavenge excess ROS has been hypothesized to differentiate susceptible genotypes from tolerant genotypes [26, 82–84]. Although cellular ROS can be generated from multiple compartments, the plasma membrane-bound reactive-burst oxidases (RBOHs) are among the first to respond to external stimuli and are a central cog in ROS-mediated signaling [85, 86]. RBOH-mediated ROS generation can activate a number of wall-bound and cytoplasmic proteins triggering diverse cellular responses. Upregulation of RBOHs, downstream signaling proteins, and upregulation of a number of genes encoding SODs (specifically Cu/Zn SODs), peroxidases, laccases, and glutathione-S-transferases (GSTs) were detected upon GB herbivory of switchgrass, indicating that both ROS signaling and potentially an increased need to modulate ROS levels had occurred in the GB-infested plants. Cu/Zn SODs, which can be localized in multiple compartments including cell walls and the cytoplasm [87], were expressed more highly in the infested plants indicating a possibility of ROS mitigation across several cellular compartments. Similar observations have been made in other plants [2, 80]. Ascorbate peroxidases and class III secreted peroxidases are among the major ROS detoxifying enzymes in switchgrass cells [88]. Four genes encoding cytosolic ascorbate peroxidases were significantly upregulated in GB-infested plants, and orthologs have been linked to wound responses [86]. Peroxidases also have well established roles in defensive responses to herbivores [88–90], and laccases, frequently associated with cell wall fortification [91], presumably play similar roles in switchgrass and other related grasses [29]. ROS content and activities of peroxidases and laccases were significantly elevated in GB-infested plants 10-DAI, supportive for an overall role of ROS, and ROS mitigation in switchgrass plants infested with GB. These data are consistent with many literature reports cited elsewhere in the text.

### Hormone defense signaling pathways

The plant hormones SA, JA, and ET play key roles as signaling molecules during both abiotic and biotic stresses, including plant-aphid interactions [92–95]. SA biosynthesis and SA-dependent pathways can be induced by phloem-feeding aphids and spider mites [96], and SA has been shown to act as a negative modulator of the JA pathway, but it can also act additively or synergistically, depending on the system [97]. In susceptible soybean plants, aphids depress both JA and SA-mediated defense responses by activating abscisic acid (ABA)-related pathways [27]. In contrast, GB herbivory elicited large-scale defensive responses in Summer switchgrass that included upregulation of genes involved in SA, JA, and ET biosynthesis (Fig. 4a–c) and downstream targets of these phytohormones including *PR* genes (Fig. 5a).

The JA metabolism associated genes, including *LOX*, *AOS*, *AOC*, and *JAR*, were shown to be upregulated in response to wounding, insect feeding, as well as necrotrophic pathogens [96, 98–101]. With the exception of three *JAR* genes which were downregulated, all other JA pathway genes were significantly induced in GB-infested switchgrass (Fig. 4b) which is consistent with other studies [96]. JA has been shown to regulate plant growth and development, and its down-regulation has been linked to an increase in susceptibility during insect infestations [1]. Therefore, the upregulation of these genes suggests that a heightened level of defense could be linked to the tolerant behavior of the switchgrass cultivar Summer. However, more work will need to be completed to clarify whether these induced genes were specifically upregulated in response to GB or part of a broader suite of plant defenses that respond to piercing-sucking insects or biotic stresses in general.

The apparent simultaneous upregulation of genes associated with SA and JA in plants subjected to aphid herbivory have been reported in the literature [102–105]. It is conceivable that in switchgrass, at least for cultivar Summer x GB interactions, associations between SA, JA, and ET are additive or synergistic. Ultimately, similar studies on switchgrass responses with diverse aphid or other arthropod pests should unravel the commonality or uniqueness of the interactions between SA, JA, and ET pathways in host plant defense.

### Anti-nutritional genes and metabolites are induced during GB infestation

Consistent with gene expression profiles, the broad scale defensive response of Summer switchgrass plants to GBs was also confirmed by metabolites produced in response to herbivory, which included pipecolic acid, trehalose, and chlorogenic acid.

Pipecolic acid is important to plant defense including aphid herbivory [54, 106, 107] and is a known signaling compound required for systemic acquired resistance (SAR) [108–110]. Likewise chlorogenic acid is associated with plant defense [55] and can negatively affect insect health [111]. Trehalose can regulate carbohydrate metabolism in leaves [112, 113], and its levels increase in response to insect herbivory [24, 53]. In Arabidopsis, trehalose also regulates *PHYTOALEXIN DEFICIENT 4* (*AtPAD4*) expression and changes the flux of glucose towards starch synthesis and away from sucrose synthesis, depriving aphids of accessible energy sources [53]. *AtPAD4* orthologs in switchgrass were not differentially expressed between controls and infested plants across all harvest dates which could be consistent with downregulation of genes associated with both starch and sucrose synthesis in GB-infested switchgrass plants. The patterns of regulation of genes associated with primary metabolism appear to be both plant and herbivore dependent, and transcriptomic events observed in this current study should be interpreted within the framework suggested by Zhou et al. [114].

Transcript evidence also supported the upregulation of enzymes involved in the glyoxylate cycle in GB-infested switchgrass plants. These included two isocitrate lyases (*Pavir.Ba00758, Pavir.Bb02888*), two malate synthases (*Pavir.Gb01372, Pavir.J04298*), and one malate dehydrogenase (*Pavir.Aa03554*). The glyoxylate cycle is upregulated during switchgrass leaf senescence [34] and has also been shown to part of plant-microbe interactions [115]. Therefore, glyoxylate metabolism could be another important aspect of herbivore defense as has been predicted for other pathogens or trigger defense-associated senescence [115–117].

## Conclusions

### A model of switchgrass responses to GB

Based on our datasets, a model underlying switchgrass response to GB feeding is proposed (Fig. 9). Among the earliest transcriptional changes occurring 5-DAI were related to a number of cell wall receptors, including wall-associated kinases. These changes appear to be similar to other studies reported in the literature (for example [7]). Perception of GB likely triggered intracellular signaling potentially through an upregulation of RBOHs and other wall-anchored proteins. Expression changes of genes linked to cell wall structure and glycans and to JA, SA, and ET biosynthesis and signaling were also induced 5-DAI. ROS levels, peroxidase and laccase activities were significantly higher by 10-DAI in GB-infested plants, and were accompanied by a massive upregulation of genes, including NACs, WRKYs, and MYBs, and potential ancillary signaling molecules such as leucine aminopeptidases (LAP, [94]). The net result appears to be a broad scale defensive response, starting from downregulation of primary metabolism to potentially starve GB of nutrients and minerals, to the production of defense metabolites and cell wall fortification (possibly through ectopic lignification), and the induction of a number of cytochrome P450s, terpene cyclases, and several dioxygenases at 10-DAI. Interestingly, both terpene cyclases and dioxygenases are important for plant defense against insects [118, 119].

These strong defensive responses observed at 10-DAI were followed by an apparent recovery of leaf functions related to photosynthesis, C, N, and nutrient metabolism 15-DAI, as detected by higher expression levels of genes associated with several of these pathways compared to 10-DAI. Future transcriptome-scale comparisons with resistant, susceptible, and tolerant genotypes will be necessary to conclusively link these pathways to possible routes of host resistance in switchgrass and other grasses. Overall, these studies provide new information and genes that could be useful for the continued improvement of warm-season temperate C4 perennial biomass grasses in response to herbivory.

## Additional files

Additional file 1: Table S1. Mapping statistics for RNA Seq (XLSX 12 kb)
Additional file 2: Table S2. Gene identities and primers used for RNA-Seq validation (XLSX 11 kb)
Additional file 3: Data S1 GO enrichment results (XLSX 12 kb)
Additional file 4: Data S2 Gene identity and expression profiles for data shown in Figures (XLSX 909 kb)
Additional file 5: Figure S1. Metabolite levels (a) chlorophyll, and (b) ROS (H_2_O_2_) and enzyme activities (c) peroxidases and (d) laccases in plants harvested at 10 DAI. Data are the means of triplicate assays from extracts of 5 individual plants from each treatment (control; blue bars); (infested; orange bars). (EPS 389 kb)
Additional file 6: Figure S2. Expression profiles of co-expression modules associated with Fig. 8. (EPS 3010 kb)

## Abbreviations

AAT: Aspartate aminotransferase 4
ABA: Abscisic acid
ACO: ACC oxidase
ACS: Acetyl-CoA synthetase
ALD1: LL-diaminopimelate aminotransferase/aldehyde dehydrogenase
ALDH: L-aminoadipate semialdehyde dehydrogenase
AOC: Allene oxide cyclase
AOS: Allene oxide synthase
AsnSyn: Asparagine synthetase
CA: Carbonic anhydrase
CAO: Chlorophyllide a oxygenase
CBR: Chlorophyll(ide) b reductase
CHL2: Chlorophyllase 2
CHLM: Magnesium-protoporphyrin IX methyltransferase
ChlSyn: Chlorophyll synthase
CLH: Chlorophyllase 1
CRD: Magnesium-protoporphyrin IX monomethyl ester [oxidative] cyclase
DAI: Days after infestation
DEGs: Differentially expressed genes
ET: Ethylene
F16bPase: Fructose-1,6-bisphosphatase I
FbPA: Fructose bisphosphate aldolase
FER: FERONIA
fNiR: Ferredoxin-dependent nitrite reductase
G1PAT: Glucose-1-phosphate adenylyltransferase
G3PDH: Glyceraldehyde 3-phosphate dehydrogenase
GB: Greenbug
GlnSyn-1: Glutamine synthetase
GlnSyn-2: Cytosolic glutamine synthetase
GluDHa: NADP + −dependent glutamate dehydrogenase
GluDHb: NAD + −dependent glutamate dehydrogenase
GluSynA: Ferrodoxin-dependent glutamine-2-oxoglutarate amino transferase
GluSynB: Glutamate synthase (Ferrodoxin)
GO: Gene Ontology
GST: Glutathione-S-transferase
HCT: Hydroxycinnamoyl CoA-shikimate/quinate hydoxycinnamoyl transferase
HERK-1: HERCULES RECEPTOR KINASE 1
Inv: Invertase
JA: Jasmonic acid
JAR: Jasmonic acid-amido synthetases
JAZ: Jasmonate-ZIM domain
LKR/SDH: Lysine ketoglutarate reductase/saccharophine dehydrogenase
LOX: Lipoxygenase
MAMP: Microbe-associated molecular pattern
MDH: Malate dehydrogenase
MgC: Magnesium chelatase
NADP-ME: NADP-Malic enzyme
NR: Nitrate reductase
PAD4: PHYTOALEXIN DEFICIENT 4
PAO: Pheophorbide A oxidase
PCA: Principal component analysis
PCB2: Divinyl chlorophyllide a 8-vinyl-reductase
PEPC: Phosphorylation by phosphoenolpyruvate carboxylase
PEPcK: PEPcarboxylase kinase
PEPCK: Phosphoenolpyruvate carboxykinase
PGK: Phosphoglycerate kinase
PI: Proteinase inhibitor
POR: Protochlorophyllide reductase
PPDK: Pyruvate, phosphate dikinase
PPDK-RP: Pyruvate, phosphate dikinase regulatory protein
PR: Pathogenesis responsive
PRK: Phosphoribulokinase
R5PI: Ribose 5-phosphate isomerase A
RBOH: Reactive-burst oxidase
RGH: Resistance gene homologs
ROS: Reactive oxygen species
RP3E: Ribulose phosphate 3 epimerase
Rubisco-SSU: Rubisco-Small Subunit
SA: Salicylic acid
SAG: Senescence associated gene
SAR: Systemic acquired resistance
SBE: Starch branching enzyme
SbPase: Sedoheptulose-bisphosphatase
SOD: Superoxide dismutase
SOX: Sarcosine (pipecolic acid) oxidase
StarP: Starch phosphorylase SucPase, Sucrose-phosphatase
StarSyn: Starch synthase
SucPSyn: Sucrose-phosphate synthase
SucSyn: Sucrose synthase genes
TBL: Trichome birefringence like
TK: Transketolase
TOM: Topological overlap measure
TPI: Triosephosphate isomerase
WGCNA: Weighted gene co-expression network analysis
